# The predictors of sleep quality in mothers of children with autism spectrum disorders in the west of Iran: A path analysis

**DOI:** 10.1016/j.heliyon.2024.e41136

**Published:** 2024-12-11

**Authors:** Ensiyeh Jenabi, Azam Maleki, Erfan Ayubi, Saeid Bashirian, Mahdieh Seyedi, Sara Abdoli

**Affiliations:** a Autism Spectrum Disorders Research Center, Hamadan University of Medical Sciences, Hamadan, Iran; b Social Determinants of Health Research Center, Health and Metabolic Diseases Research Institute, Zanjan University of Medical Sciences, Zanjan, Iran; c Social Determinants of Health Research Center, Hamadan University of Medical Sciences, Hamadan, Iran; d Hamadan University of Medical Sciences, Hamadan, Iran

**Keywords:** Sleep quality, Children, Autism spectrum disorders

## Abstract

There is limited data available on the impact of sleep problems in children with ASD on parents' sleep quality. Due to the lack of research in Iran on factors affecting the sleep quality of mothers of children with ASD, this study was designed to explore predictors of mothers' sleep quality using path analysis. From October 2022 to May 2023, a cross-sectional study was conducted in Hamadan, a city in western Iran, involving 100 mothers of children with ASD. Data were collected using a demographic checklist and four questionnaires were included Perceived social support, Petersburg Sleep Quality Questionnaire (PSQI), Children's Sleep Habits Questionnaire (CSHQ), and Perceived Stress. Data analysis was performed using SPSS Version 16, and path analysis was conducted with LISREL software version 8.5. Statistical significance was determined by P-values less than 0.05. The sleep quality of mothers had no significant relationship with any of the demographic variables (p > 0.05). Correlation bivariate analysis showed that the total score of Sleep Quality of mothers had a positive significant correlation with Perceived Stress (r=0.28) and Sleep Habit of Children (r=0.51) but had a negative significant correlation with Social Support (r=−0.31). Children's sleep habits, perceived stress, and perceived social support are the main predictors of Sleep Quality in Mothers of children with autism spectrum disorders. Our study showed sleep quality in mothers of children with ASD may be the function of the child's sleep pattern, social support, and stress. We recommended that our hypothesized model should be enriched with more covariates and modifiers and also be tested in further large-scale prospective studies.

## 1 Introduction

Autism spectrum disorder (ASD) is a neurodevelopmental condition marked by challenges in social communication and repetitive, stereotyped behaviors and interests [1]. This disorder is becoming more prevalent globally. According to estimates from the CDC's Autism and Developmental Disabilities Monitoring (ADDM) Network, about 1 in 36 children is affected by ASD [2].

There is limited information about the prevalence of ASD in Iran. A study reported that the ASD prevalence is 6.26 per 10,000 among five-year-old children in Iran [3]. The ASD diagnostic assessment methods, such as the Gilliam Autism Rating Scale (GARS) and the Autism Diagnostic Interview-Revised (ADI-R), have undergone psychometric evaluation in Iran [4,5].

Sleep disorders include problems in the quality, timing, and amount of sleep that led to daytime distress and impaired functioning. Sleep-wake disorders often co-occur with medical or other mental health conditions such as depression, anxiety, or cognitive impairment [6]. Insomnia disorder can be classified into three categories according to the International Classification of Sleep Disorders-3rd Edition (ICSD-III). These categories encompass chronic insomnia disorder, short-term insomnia disorder, and other forms of insomnia disorder [7]. The ICSD-III also outlines different subtypes of insomnia. For example, psychophysiological insomnia is characterized by increased arousal and worry about sleep, making it difficult to sleep at home but easier in other environments. Idiopathic insomnia is a lifelong condition that begins in childhood and may be related to genetic or congenital factors. Paradoxical insomnia involves underestimating the amount of sleep obtained and perceiving periods of wakefulness as sleep. Inadequate sleep hygiene refers to poor sleep habits and behaviors, such as excessive daytime napping or using electronic devices before bed. Behavioral insomnia of childhood refers to sleep issues in children related to dependencies on certain stimuli or disruptions in routines. These classifications and subtypes help in understanding and diagnosing different manifestations of insomnia [8].

Sleep problems are prevalent in children with ASD, affecting between 33 % and 80 % of this population [9]. The underlying mechanisms of sleep disorders in children with autism are still not fully understood. Considering the vital functions of sleep in promoting brain connectivity, neural plasticity, emotional regulation, and social behavior,all critical aspects in ASD pathogenesis, synaptic dysfunction emerges as a major mechanism linking ASD and sleep disorders [10].

Sleep disturbances are one of the most demanding problems for caregivers with child ASD [11]. Different studies have indicated that sleep problems are more common among children with ASD and their parents compared to non-autistic children and their parents [12,13]. Bin Eid et al. reported that among children with ASD, both mothers and children were likely to experience sleep deprivation, characterized by shorter sleep duration. Moreover, they were prone to poorer sleep quality, evident in longer sleep onset latencies and increased frequencies of wake bouts. The study also revealed that the child's sleep behaviors, such as the presence of sleep-disordered breathing and the duration of wake bouts, significantly influenced the quality of mothers' sleep. This provides preliminary evidence of the interaction between mothers' and children's sleep habits in the context of ASD [14].

Studies have demonstrated that maternal sleep quality is a crucial predictor of maternal depression, stress, and fatigue [15]. Sleep issues among children with developmental disabilities are linked to greater behavioral problems and heightened stress levels in mothers [16]. Social support acts as a protective factor, reducing maternal stress and improving sleep quality [17]. This aligns with the stress-buffering hypothesis, which suggests social support mitigates the impact of stress on health [17].

Varma et al. (2021) conducted a systematic review and reported that sleep quality, insomnia symptoms and measured sleep duration and sleep efficiency were related between parents and their children. In addition, children's sleep disturbances are associated with poorer sleep quality, more insomnia symptoms, and increased parental arousal pre-sleep [18]. Leader et al. (2022) investigated the relationship between sleep and factors like stress, anxiety, depressive symptoms, social support, and quality of life in parents of children with Autism Spectrum Disorder (ASD). Their study, involving nine children with ASD and their parents, found that mothers of children with ASD experienced significant sleep problems associated with elevated stress, anxiety, and depressive symptoms. Furthermore, children with ASD demonstrated lower levels of social support [19].

Mihaila and Hartley reported that persistent poor sleep quality was associated with inter-individual differences in mothers' initial ratings of the level of behavior problems of children with ASD [20]. The Path analysis study by Liu et al. (2021) showed that sleep problems in ASD children had both direct and indirect effects on maternal parental physical health summary scores. They indicated that sleep problems in children with ASD may negatively affect parents' quality of life, and act as independent influencing factors on parents' physical health [21].

In Iran, most specialized services needed by children with ASD, such as speech and occupational therapies, along with family training programs, are not typically provided by public centers or clinics. Instead, these services are offered by independent private organizations associated with welfare organizations. Moreover, health insurance does not cover ASD rehabilitation services [22]. This study is novel in its focus on maternal sleep quality, examining how it is influenced by child sleep behaviors and factors like social support and stress in the context of Iran, where research on this topic is limited. It integrates social support as a mediating factor, offering new insights into its impact on maternal sleep. Using path analysis, the study provides a comprehensive view of the complex relationships between maternal and child sleep, considering the cultural and healthcare challenges in Iran.

Hypothesis.

H1 Children's sleep habits directly and indirectly affect the sleep quality of their mothers through perceived stress.

H2 Social support influences mothers' sleep quality by affecting perceived stress and children's sleep habits.

H3 Perceived stress mediates the relationship between social support, the sleep habits of children, and the sleep quality of mothers.

## 2 Methods

### 2.1 Setting and design of the study

The current cross-sectional study was conducted on 100 mothers who have a child with autism spectrum disorders in Hamadan, a city in the west of Iran, from October 2022 to May 2023. Mothers of children with ASD refer to the Autism Spectrum Disordered Research Center to receive rehabilitation services for their children, and all children had documents in the center. Written consent was obtained from the mothers of children with ASD to participate in the intervention. Mothers completed the questionnaires themselves. Children with level 2 (requiring substantial support) were included in this study. Moreover, these children were diagnosed with ASD according to the DSM-5 criteria and the ADI-R. Additionally, the diagnosis was confirmed by a psychiatrist.

### 2.2 Conceptual model

**Fig. 1:**
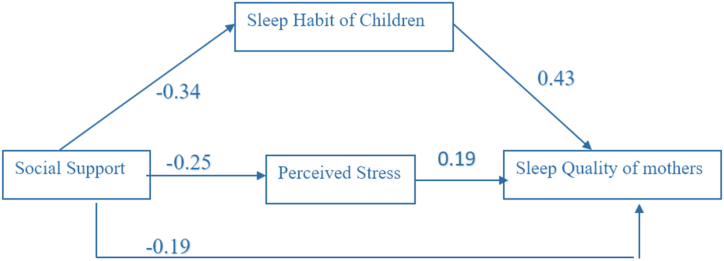
The conceptual model of the effects of perceived social support, children's sleep habits, and perceived stress on sleep quality of women.

The conceptual model is presented in Fig. 1. A path model was utilized to examine the causal relationships and assess the direct, indirect, and total effects of the independent variables (Perceived Social Support, Children's Sleep Habits, and Perceived Stress) on the dependent variable (Sleep Quality). Initially, a conceptual model (Fig. 1) was developed to quantify these effects of perceived social support, children's sleep habits, and perceived stress on Sleep Quality specifically in women who have a child with autism spectrum disorders. This conceptual model, entering selected variables, and defining relationships between them was based on logical and theoretical analyses. In this conceptual model, we considered Perceived social support as mediators, Sleep Quality as a dependent variable, as well as Perceived stress and children's sleep habits as independent predictors. The path model extends beyond simple regressions by enabling the evaluation of multiple dependent variables simultaneously. It also allows variables to act as both dependent and independent concerning different variables within the model.

### 2.3 Sample size and sampling method

Based on a power analysis considering a moderate effect size (0.15, Cohen's d), predicting three variables with a confidence level of 95 % and a Power (1-β err prob) =0.85, the calculated sample size was 87 individuals. However, considering a potential 10 % dropout rate, the sample size can be increased up to 100 individuals. The sample size included all eligible mothers of children with ASD registered at the Research Center of Autism Spectrum Disorders affiliated with Hamadan University of Medical Sciences (100 people), selected through the convenience sampling method. In this study, due to the availability of records of all children with autism, random sampling methods could not be used.

### 2.4 Inclusion and exclusion criteria

Inclusion criteria were as follows: 1. Mothers who have a child with autism spectrum disorders 3–12 years old, 2. Autism diagnosis has been confirmed based on DSM-5 and the ADI-R autism diagnostic interview. 3. Speak in the Persian language and be literate. Exclusion criteria were included 1. The mothers suffering from chronic physical diseases (having heart disease, arthritis, diabetes, stroke and lung diseases) according to the patient self-report, 2. The mothers sufferd from a known mental illness, 3. Mother was taking psychotropic medication and 4. The child or parent has been diagnosed with obstructive sleep apnea, narcolepsy, or restless legs syndrome. 5. The children included in this study were not using anti-epileptic and sedative drugs.

### 2.5 Measurements

Data were collected using a demographic checklist and four questionnaires were included Perceived social support, Pittsburgh Sleep Quality Index (PSQI), Children's Sleep Habits Questionnaire (CSHQ), and Perceived Stress.

#### 2.5.1 Demographic checklist

It included the child's sex, child's age, child's birth rank, mothers' age, mothers' age at the time of delivery of the child, mothers' education, and mothers' job.

#### 2.5.2 Multidimensional scale of perceived social support

The Multidimensional Scale of Perceived Social Support consists of 12 questions on a 5-point Likert grading from 1: strongly disagree to 5: strongly agree. These questions are designed to assess an individual's perception of social support from three different sources: family, friends, and significant others. e.g., “My family really tries to help me", "I get the emotional help and support I need from my family", and etc. A higher score indicates more social support. The total score was classified into three levels. A total score of 1–2.33 indicates low social support, scores of 2.34–3.67 indicate moderate support and a score of 3.68–5 indicates high social support [23]. The psychometric properties of the Persian version of the questionnaire were tested by Bagharian [24]. The reliability of the questionnaire was stated to be 84 %. In this study, the Cronbach's alpha coefficient of the scale was 0.91.

#### 2.5.3 The Pittsburgh Sleep quality index (PSQI)

The Pittsburgh Quality Sleep Questionnaire, developed by Buysse et al. [25], evaluates sleep quality over the past month across seven components: subjective sleep quality, sleep latency, sleep duration, habitual sleep efficiency, sleep disturbances, use of sleeping medication, and daytime dysfunction. The first four components are assessed descriptively, while the remaining questions are multiple-choice. Responses to multiple-choice questions are scored on a Likert scale from zero to three: one point for less than once a week, two points for once or twice a week, and three points for three or more times a week. The total score ranges from zero to 21, with scores above five indicating poor sleep quality.

#### 2.5.4 Children's Sleep Habits Questionnaire (CSHQ)

The original version of the Children's Sleep Habits Questionnaire, created by Owens et al. [26], is intended for children ages 4–10. It comprises 45 items, with 33 items used to derive scores for eight subscales as well as a total sleep disturbances score, all completed by parents. The questionnaire is categorized into eight subscales: 1) Bedtime Resistance, 2) Sleep Onset Delay, 3) Sleep Duration, 4) Sleep Anxiety, 5) Night Waking, 6) Parasomnia, 7) Sleep-Disordered Breathing, and 8) Daytime Sleepiness. Each item is scored on a Likert scale from 1 to 3, reflecting the frequency from rarely to usually. The score range for the Children's Sleep Habits Questionnaire, based on its 45 items and the Likert scale scoring from 1 to 3, totals between 33 and 99 points. The total score is determined by adding up the scores from all the subscales, with each subscale score being the sum of the scores of its related items. Higher scores on the Children's Sleep Habits Questionnaire indicate greater sleep difficulties. In a non-clinical sample of children aged 4–10 years, the Cronbach's alpha coefficient for the subscales was 0.70. The reliability estimation by the test-retest method with a two-week interval was in the range of 0.62–0.79. The psychometric properties of the Persian version of the tool were evaluated by Shoghy and its reliability was evaluated by the retest method with a two-week interval in the case of 10 children aged 6–11 years, 0.97 was determined [27]. In this study, the Cronbach's alpha coefficient for the scale was 0.81.

#### 2.5.5 The Perceives Stress Scale (PSS-10)

The Perceives Stress Scale (PSS-10) consists of 10 items with a 5-point Likert scoring from never to very much, which includes stress experiences in the past month [28]. Here are a few sample questions from the PSS-10: "In the past month, how frequently have you felt unable to control the important aspects of your life?" "During the past month, how often have you felt confident in your ability to handle your personal issues?" "Over the past month, how frequently have you felt that things were going well for you?" And so on. The range of stress scores is between 0 and 40, and a higher score indicates higher perceived stress. Total scores of 13 and below indicates normal stress, 14–19 indicate intermediate stress, and a score of 20 and above indicate high stress. The psychometric properties of the questionnaire were tested by Khalili et al. in Iran and its reliability was mentioned as 72 % [29]. In this study, the Cronbach's alpha coefficient for the scale was 0.69.

### 2.6 Statistical analysis

Data analysis was performed using SPSS Version 16, and path analysis was conducted with LISREL software version 8.5. Descriptive statistics were used to analyze the data on demographic characteristics. The normality of the data was confirmed using the Kolmogorov–Smirnov test, indicating a normal distribution for all variables (P-value ≥0.05). Bivariate Pearson correlations were conducted to examine the associations among Perceived Social Support, Children's Sleep Habits, Perceived Stress, and Sleep Quality. Statistical significance was set at P-values below 0.05. Correlations above 0.4 were generally considered relatively strong, correlations between 0.2 and 0.4 were regarded as moderate, and those below 0.2 were considered weak correlations. The relationship between demographic characteristics and mothers' sleep quality was investigated using a chi-square test with a cut-off point of 5. Model fit indices, including RMSEA (Root Mean Square Error of Approximation), AGFI (Adjusted Goodness of Fit Index), CFI (Comparative Fit Index), and Chi-square/df, were employed to assess model fit. A RMSEA value below 0.07, Chi-square/df ratio less than 3, AGFI greater than 0.9, and CFI exceeding 0.95 indicated a well-fitting model. Additionally, a t-value greater than +2 or less than −2 was considered statistically significant.

## 3 Results

All mothers of children with ASD in this study responded to the questionnaires.

### 3.1 Baseline data

The mean (SD) age of mothers was 36.75(6.12) years old and the mean (SD) age of mothers at the time of delivery of the child was 30.17(6.05) years old. Out of 100 women who took part in this study, 58 % of them were in the 36–45 age groups. Fifty-three percent of them were educated at the university level. Fifty percent of Mothers were in the 26 to 35 age group at the time of delivery of the child. Most of them were housewives (81 %). Regarding the characteristics of the children of these mothers, most of the babies were boys (70 %), the age group of 6–9 years (42 %), and the first child in family (53 %) (Table 1).

**Table 1:** The frequency of mothers 'sleep quality in terms of demographic characteristics (n=100 people).

Variables		Frequency (%)	PSQI <5	b PSQI≥5	P value^a^
Child 's Sex	Male	75(75.0)	17(77.3)	58(74.4)	0.780
	Female	25(25.0)	5(22.7)	20(25.6	
Child 's Age	3–5	39(39)	10(45.5)	29(37.2)	0.769
	6–9	42(42)	8(36.4)	34(43.6)	
	10–14	19(19)	4(18.1)	15(19.2)	
Child 's Birth Rank	1	53(53.0)	12(54.5)	41(52.6)	0.924
	2	35(35.0)	7(31.8)	28(35.9)	
	3>	12(12.0)	3(13.6)	9(11.5)	
Mothers' age	17–25	5(5.0)	0	5(6.4)	0.600
	26–35	31(31.0)	7(31.8)	24(30.8)	
	36–45	58(58.0)	13(59.1)	45(57.7)	
	45>	6(6.0)	2(9.1)	4(5.1)	
Mothers' age at the time of delivery of the child	15–25	27(27.0)	6(27.3)	21(26.9)	0.999
	26–35	50(50.0)	11(50)	39(50)	
	35–43	23(23.0)	5(22.7)	18(23.1)	
Mothers' education	Elementary	7(7.0)	1(4.5)	6(7.7)	0.215
	Guidance	10(10.0)	1(4.5)	9(11.5)	
	High School	30(30.0)	4(18.2)	26(33.3)	
	University	53(53.0)	16(72.7)	37(47.4)	
Mothers' Job	Housewife	81(81.0)	16(72.7)	65(83.3)	0.263
	Employed	19(19.0)	6(27.3)	13(16.7)	

a chi-square test.

b The Pittsburgh Sleep Quality Index (PSQI).

### 3.2 The Pittsburgh Sleep quality index

The results of the chi-square test showed that the sleep quality of mothers had no significant relationship with any of the demographic variables (p > 0.05) (Table 1).

The Mean (SD) total score of Sleep Quality of mothers was 7.88 (4.20) which is more than the score of 5. The Mean (SD) total score of Perceived Stress was 22.66 (4.23) which is more than 20 and indicates high stress. The Mean (SD) total score of Social Support was 2.55 (0.82) indicating moderate to high-level social support, and The Mean (SD) total score of Sleep Habit of Children were 55.42 (9.55) (Table 2).

**Table 2:** Mean (SD), Minimum, and Maximum of perceived Stress, Social Support, Sleep Quality of mothers, and Sleep Habit of Children.

Variables		Mean	SD	Min	Max
Mothers' Sleep Quality	Subjective sleep quality	1.14	0.75	0	3
	Sleep latency	1.73	0.81	0	3
	Sleep duration	0.58	0.87	0	3
	Sleep efficiency	0.44	0.85	0	3
	Sleep disturbances	1.38	0.63	0	3
	Use of sleep medications	0.41	0.84	0	3
	Day time dysfunction	1.02	0.90	0	3
	Total score	7.88	4.20	0	20
Social Support	Total score	2.55	0.82	1	5
Perceived Stress	Total score	22.66	4.23	12	35
Children's Sleep Habits	Bedtime Resistance	11.75	2.68	6	17
	Sleep Onset Delay	1.74	0.76	1	3
	Sleep Duration	4.89	1.46	3	8
	Sleep Anxiety	7.20	2.41	4	12
	Night Waking	4.54	1.52	3	9
	Parasomnias	9.44	2.23	7	17
	Sleep Disordered Breathing	3.77	1.30	3	9
	Daytime Sleepiness	12.05	3.00	8	23
	Total score	55.42	9.55	37	84

The total score on the questionnaire ranged from zero to 21, with 78 % of participants having poor sleep quality, considering scores above five.

The total score of sleep quality in mothers showed a positive moderate significant correlation with perceived stress (r=0.28) and a high significant correlation with children's sleep habits (r=0.51). While the sleep quality of mothers had a negative moderate significant correlation with social support (r=−0.31). The children's sleep habits correlated with the mothers' perceived Social Support but did not correlate with the mothers' perceived Stress (Table 3).

**Table 3:** The correlations between the total score of perceived Stress, Social Support, Sleep Quality of mothers, and Sleep Habit of Children.

	1	2	3	4
1- Sleep Quality of mothers	1			
2- Perceived Stress	0.281^a^	1		
3- Social Support	−0.386^a^	−0.254^b^	1	
4- Sleep Habit of Children	0.519^a^	0.107	−0.341^a^	1

Values are given as Pearson coefficient (P-value) using the Pearson correlation test.

a Correlation is significant at the 0.01 level (2-tailed).

b Correlation is significant at the 0.05 level (2-tailed).

In this study, we examined the association between a mother's occupation and perceived stress level using a chi-square test. The findings suggested that these variables did not exhibit a statistically significant relationship.

The bivariate analysis revealed significant positive correlations between the CSHQ subscales and mother's sleep quality. Specifically, bedtime resistance (r=0.31, p=0.002), sleep anxiety (r=0.29, p=0.003), night waking (r=0.34, p=0.001), parasomnia (r=0.35, p=0.001), sleep disordered breathing (r=0.43, p=0.001), and daytime sleepiness (r=0.38, p=0.001) were found to be significantly correlated. However, no significant correlations were observed between sleep onset delay and sleep duration.

The Model is Saturated, the Fit is Perfect [CFI: comparative fit index=1, GFI: goodness fit index=1, RMSEA: Root Mean Square Error of Approximation=0.001, Degrees of Freedom = 1, Minimum Fit Function Chi-Square = 0.052 (P = 0.82)] (Table 4)

**Table 4:** Model fit indicators.

Indicator	Value	Standard Value
CFI (Comparative Fit Index)	1.00	≥0.90
GFI (Goodness of Fit Index)	1.00	≥0.90
RMSEA (Root Mean Square Error of Approximation)	0.001	≤0.08
Degrees of Freedom	1	–
Minimum Fit Function Chi-Square	0.052 (P = 0.82)	P ≥ 0.05

Path coefficients for total Sleep Quality are presented in Table 5. We found that the total effect of children's sleep habits on Sleep Quality of participants was 0.43 and Perceived stress was 0.19 respectively. Perceived social support has an indirect effect on Sleep Quality of participants through Perceived Stress. Perceived Stress is a mediator that predicts Sleep Quality. Therefore, children's sleep habits, perceived stress, and Perceived social support are the main predictors of Sleep Quality in Mothers of children with autism spectrum disorders (Table 5).

**Table 5:** Path coefficients for a total score of Perceived Stress, Social Support, Sleep Quality of mothers, and Sleep Habit of Children.

Predictors		Direct effect	Indirect effect	Total effect	T-value∗
Sleep Quality of Mothers	Sleep Habit of Children	0.43	–	0.43	5.87
	Perceived Stress	0.19	–	0.19	2.71
	Social Support	−0.19	−0.06	−0.25	2.13

## 4 Discussion

In this study, we found that perceived stress, sleep habit of children, and social support may be determinants of sleep quality of mothers of children with ASD. Our hypothesized model for the interrelationship nature among the studied variables showed a good fit with the data. In the model, perceived social support has an indirect effect on Sleep Quality of participants through Perceived Stress. Perceived Stress is a mediator that predicts Sleep Quality.

Regardless of the effect of social support on stress, other psychiatric symptoms such as anxiety and depression can also be influenced by social support. For example, it has been demonstrated that social support can mediate the relationship between anxiety and sleep quality of parents of children with psychiatric, neurological, and movement disorders with an indirect effect size of 5 % [30]. The mitigating role of social support on distress in parents of children with disabilities has been suggested in previous studies [31,32]. Mothers of autistic children in Iran have access to various types of support to help them in their caregiving journey. These supports include educational programs that provide information and guidance on autism, therapeutic services such as speech therapy, support groups where mothers can connect with others facing similar challenges, counseling and psychological support to address emotional well-being, and advocacy and legal support to protect their child's rights [33]. However, the availability and utilization of these supports may differ across regions. Hence, although provided support by friends and family can address distress and sleep issues in the parents, however, special attention should be paid to the main sources of stress in parents such as severe autistic symptoms and the child's behavioral and emotional impairments [34,35].

Our study provides evidence of the direct effect of the sleep habit of autistic children on the sleep quality of parents, which was consistent with previous studies [36]. Lopez et al., demonstrated that parents of children diagnosed with autism independently reported experiencing more sleep difficulties compared to parents of typically developing children [12]. Meltzer et al., found that parents of children with ASD exhibited lower sleep quality in comparison to the typically developing group. Furthermore, parents of children with ASD displayed objectively distinct sleep patterns, including earlier wake times and shorter total sleep durations, when contrasted with parents of typically developing children [37].

In the current study, a significant positive correlation was found between the CSHQ subscales and mother's sleep quality. Specifically, the subscales of bedtime resistance, sleep anxiety, night waking, parasomnia, sleep disordered breathing, and daytime sleepiness all showed significant positive correlations. However, no significant correlations were observed between sleep onset delay and sleep duration. These findings suggest that various aspects of children's sleep patterns and behaviors are related to the sleep quality experienced by their mothers. It is important to note that the absence of significant correlations between sleep onset delay and sleep duration indicates that these particular aspects of children's sleep do not appear to have a direct impact on maternal sleep quality in this study. Further research is necessary to explore potential factors contributing to this lack of correlation and to achieve a more comprehensive understanding of the relationship between children's sleep and the quality of maternal sleep.

Since the sleep problems among autistic children are well established in the previous studies [[38], [39], [40]], an important point that can be considered in the mind is that attention to the predisposing factors of sleep hygiene practices and sleep quality in autistic children can help to improve the sleep quality of their parents. For example, some factors such as regulating screen time before sleep and thermal comfort during sleep [41] and social interaction difficulties [42] have been suggested in previous studies as determinants of sleep quality in autistic children. The causal pathway between child sleep quality and parent sleep quality can be enriched by adding more covariates. For example, in the suggested model in one study [43], child sleep can be the function of ASD-related major adversities and caregiver impression of severity, and also caregiver strain was a modifier variable between child sleep and caregiver sleep.

In this study, baseline data was collected on the characteristics of the participating mothers and their children. The average age of the mothers was 36.75 years. The majority of the mothers (81 %) were housewives. In terms of the children, 70 % of them were boys, 42 % were in the 6–9 age group, and 53 % were the first child in their family. Additionally, most of the mothers had a university education and were also housewives. The results of the current study aligned with those of Mihaila et al. [20]. Indeed, in their study, they reported the characteristics of fathers as well. The widespread occurrence of sleep problems in children with ASD, coupled with the adverse effects of inadequate sleep on mothers, highlights the critical necessity for targeted interventions in numerous households. Current literature can guide clinicians, indicating that promoting positive sleep hygiene alongside standard extinction techniques is likely to yield improvement. Addressing difficulties in initiating and maintaining sleep in children with ASD, as well as implementing scheduled awakenings, may help alleviate this condition.

### 4.1 Limitations

The study findings should be interpreted cautiously due to several limitations. The cross-sectional nature of the study may obscure the true temporal relationships among the variables. Limitations include a small sample size, convenience sampling, lack of information on the severity of ASD symptoms in caregivers, and self-report data that may introduce measurement bias. Additionally, the study lacked objective measures of sleep and did not investigate specific types of sleep medication used by mothers, which could affect generalizability. Stressors like behavioral outbursts and violence in children with ASD were not addressed, further impacting the study's generalizability. However, in the present study, fathers did not participate, and this was considered as a limitation.

### 4.2 Practical implications

Future research could benefit from designing longitudinal studies that track mothers of children with ASD over an extended period. This approach would allow researchers to examine how changes in children's sleep habits, perceived stress levels, and social support impact mothers' sleep quality over time. By collecting data at multiple time points, researchers could explore the temporal dynamics of these relationships and identify potential moderating or mediating factors that could influence the associations found in the current study. Furthermore, while the present study focused exclusively on mothers, future research should also include fathers to explore the role of paternal support in enhancing maternal well-being. Investigating fathers' contributions could provide a more comprehensive understanding of family dynamics and support systems. Additionally, incorporating qualitative, open-ended questions into surveys could offer valuable insights into the complex factors influencing maternal sleep quality. This approach has the potential to reveal deeper, context-specific influences on mothers' sleep patterns that may not be captured through quantitative measures alone.

## 5 Conclusion

In conclusion, our study showed sleep quality in mothers of children with ASD may be the function of the child's sleep pattern, social support, and stress. We recommended that our hypothesized model should be enriched with more covariates and modifiers and also be tested in further large-scale prospective studies. Also, the need to carry out interventions to improve children's sleep, increase social support and reduce stress in mothers of children with ASD, which can be lead to the improvement of mothers' sleep quality, seems necessary.

## CRediT authorship contribution statement

**Ensiyeh Jenabi:** Writing – review & editing, Writing – original draft, Visualization, Resources, Project administration, Investigation, Funding acquisition, Data curation, Conceptualization. **Azam Maleki:** Writing – review & editing, Writing – original draft, Visualization, Validation, Methodology, Formal analysis, Data curation. **Erfan Ayubi:** Writing – review & editing, Writing – original draft, Software, Methodology, Investigation, Data curation. **Saeid Bashirian:** Writing – review & editing, Writing – original draft, Visualization, Validation, Supervision, Conceptualization. **Mahdieh Seyedi:** Writing – review & editing, Writing – original draft, Validation, Software, Resources, Investigation, Data curation. **Sara Abdoli:** Writing – review & editing, Writing – original draft, Visualization, Validation, Software, Investigation, Data curation, Conceptualization.

## Consent for publication

Not applicable.

## Ethics approval and consent to participate

The study protocol was accepted by the ethical committee of Hamadan University of Medical Sciences with code IR.UMSHA.REC.1401.438. Informed consent was obtained from all individual participants included in the study. We performed this study in accordance with the Declaration of Helsinki.

## Data availability

The authors confirm that the data supporting the findings of this research are available within the article. The dataset used in the present study is available from the corresponding author upon reasonable request.

## Funding

This study was supported by 10.13039/501100004697Hamadan University of Medical Sciences with Code: IR.UMSHA.REC.1401.658.

## Declaration of competing interest

The authors declare that they have no known competing financial interests or personal relationships that could have appeared to influence the work reported in this paper.

## Abbreviations

CSHQ: Children's Sleep Habits Questionnaire
PSQI: Petersburg Sleep Quality Questionnaire
ASD: Autism spectrum disorder
ICSD-III: Classification of Sleep Disorders-3rd Edition
ADDM: Autism and Developmental Disabilities Monitoring
